# A novel de novo *TP63* mutation in whole‐exome sequencing of a Syrian family with Oral cleft and ectrodactyly

**DOI:** 10.1002/mgg3.2179

**Published:** 2023-04-18

**Authors:** Claire L. Simpson, Danielle C. Kimble, Settara C. Chandrasekharappa, NISC Comparative Sequencing Program, Khalid Alqosayer, Emily Holzinger, Blake Carrington, John McElderry, Raman Sood, Ghiath Al‐Souqi, Hasan Albacha‐Hejazi, Joan E. Bailey‐Wilson

**Affiliations:** ^1^ Computational and Statistical Genomics Branch, National Human Genome Research Institute National Institutes of Health Baltimore Maryland 21224 USA; ^2^ Department of Genetics, Genomics and Informatics University of Tennessee Health Science Center Memphis Tennessee 38163 USA; ^3^ Cancer Genetics and Comparative Genomics Branch, National Human Genome Research Institute National Institutes of Health Bethesda Maryland 20814 USA; ^4^ NIH Intramural Sequencing Center, National Human Genome Research Institute National Institutes of Health Bethesda Maryland 20814 USA; ^5^ Prime Health Clinic Jeddah Riyadh 21511 Saudi Arabia; ^6^ Zebrafish Core, National Human Genome Research Institute National Institutes of Health Bethesda Maryland 20892 USA; ^7^ Hejazi Clinic P.O. Box 2519 Jeddah Riyadh 11461 Saudi Arabia

**Keywords:** cleft lip, cleft palate, limb deformities, congenital, whole‐exome sequencing

## Abstract

**Background:**

Oral clefts and ectrodactyly are common, heterogeneous birth defects. We performed whole‐exome sequencing (WES) analysis in a Syrian family. The proband presented with both orofacial clefting and ectrodactyly but not ectodermal dysplasia as typically seen in ectrodactyly, ectodermal dysplasia, and cleft lip/palate syndrome‐3. A paternal uncle with only an oral cleft was deceased and unavailable for analysis.

**Methods:**

Variant annotation, Mendelian inconsistencies, and novel variants in known cleft genes were examined. Candidate variants were validated using Sanger sequencing, and pathogenicity assessed by knocking out the *tp63* gene in zebrafish to evaluate its role during zebrafish development.

**Results:**

Twenty‐eight candidate de novo events were identified, one of which is in a known oral cleft and ectrodactyly gene, *TP63* (c.956G > T, p.Arg319Leu), and confirmed by Sanger sequencing.

**Conclusion:**

*TP63* mutations are associated with multiple autosomal dominant orofacial clefting and limb malformation disorders. The p.Arg319Leu mutation seen in this patient is de novo but also novel. Two known mutations in the same codon (c.956G > A, p.(Arg319His; rs121908839, c.955C > T), p.Arg319Cys) cause ectrodactyly, providing evidence that mutating this codon is deleterious. While this *TP63* mutation is the best candidate for the patient's clinical presentation, whether it is responsible for the entire phenotype is unclear. Generation and characterization of *tp63* knockout zebrafish showed necrosis and rupture of the head at 3 days post‐fertilization (dpf). The embryonic phenotype could not be rescued by injection of zebrafish or human messenger RNA (mRNA). Further functional analysis is needed to determine what proportion of the phenotype is due to this mutation.

## Graphical abstract

A novel de novo mutation in the *TP63* gene was found in a patient with an orofacial cleft and ectrodactyly. We showed that mutating this gene in zebrafish caused a severe phenotype where the head ruptured and we were unable to rescue the phenotype upon injection of either zebrafish or human mRNA. It is unknown whether the mutation is responsible for the entire phenotype or just the ectrodactyly and further research is warranted.

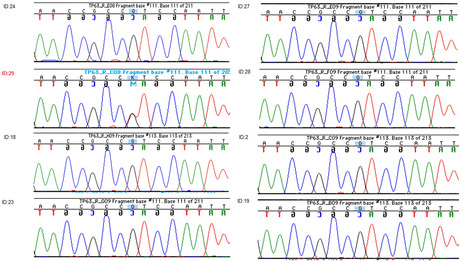

## 1 BACKGROUND

In mammalian development, the head is one of the most complex structures to form. The tissues develop from endoderm, mesoderm, ectoderm, and cranial neural crest cells, and the regulation of growth and differentiation is controlled by signaling between different cellular components both spatially and temporally in a highly complex process that can be easily disrupted (Abramyan, 2019; Murillo‐Rincón & Kaucka, 2020). This intricate interplay of numerous factors means that cleft lip, with or without cleft palate, is a clinically and genetically heterogeneous trait with multiple genes and regions mapped. There are over 400 syndromes that have orofacial clefts as a key feature (Fryns & de Ravel, 2002), but the majority of patients born with an oral cleft are non‐syndromic, where the cleft is the only malformation in the child. Only a small proportion of causal genes have been identified for either syndromic or non‐syndromic oral clefts.

Ectrodactyly (also known as split hand/split foot malformation, SHFM) is another clinically heterogeneous malformation (Elliott et al., 2005; Elliott & Evans, 2006) in which the absence of the central rays produces a deep median cleft in the autopod, one of the skeletal elements of the developing limb. Like oral clefts, ectrodactyly can occur as an isolated entity or as part of a syndrome. During development, gradients of signaling molecules in three spatial directions control the patterning of the limbs. Three specialized cell clusters control this process through differentiation and proliferation; the apical ectodermal ridge (AER), the progress zone, and the zone of polarizing activity. Both genetic and environmental risk factors are known to disrupt the function of the AER and cause ectrodactyly. Mutations in *TP63* (Bernardini et al., 2008; Duijf et al., 2003; Roberts & Tabin, 1994; Wang et al., 2014) and *WNT10B* (Bui et al., 1997) have been associated with ectrodactyly, and other regions of the genome have been mapped as containing some still unidentified causal genes. Duplication of 10q24 is also associated with ectrodactyly and is the most common cause of SHFM in humans, accounting for approximately 20% of cases (Klopocki et al., 2012). There are several other SHFM and oral cleft disorders, some of which include both, such as ectrodactyly, ectodermal dysplasia, cleft lip/palate syndrome‐1 (EEC1), and ectrodactyly, ectodermal dysplasia and cleft lip/palate syndrome‐3 (EEC3) which are autosomal dominant disorders that include SHFM as well as skin anomalies.

Whole‐exome sequencing (WES) has successfully identified the causal variants in a range of Mendelian diseases. Here we present the results of a WES study of a single Syrian family with a child affected with both orofacial cleft and ectrodactyly.

## 2 METHODS

### 2.1 Recruitment and clinical features

A collaborative study of familial orofacial clefts, with an emphasis on non‐syndromic oral clefts but expanded to include syndromic oral clefts, was instituted in the Syrian Arab Republic in 1998 by investigators at the National Human Genome Research Institute, National Institutes of Health, USA, and clinicians at the Ibn Al‐Nafees Hospital, Damascus, Syria (Marazita et al., 2004; Wyszynski et al., 2003). Families were ascertained through at least one individual affected with non‐syndromic cleft lip with or without cleft palate. Of these families, those with two or more family members affected with orofacial clefts were invited to enroll in this genetic study. The study was approved by the Institutional Review/Ethics Boards of the National Human Genome Research Institute, NIH (USA), and the Ibn Al‐Nafees Hospital (Damascus, Syrian Arab Republic). All study participants provided written informed consent (in Arabic), and the study followed the tenets of the Declaration of Helsinki. The informed consent forms and the protocol on file with the Institutional Review Board at the NIH both guarantee the pedigrees will never be published to protect the privacy of the study participants because these pedigrees are readily identifiable given the rarity of such multiplex oral cleft families. Therefore, only a redacted and disguised version of the pedigree is shown here. Subjects enrolled in this study were all examined by the same local physicians and were subjected to standardized interviews.

The ascertainment of all families followed the clinical guidelines proposed by the International Consortium for Oral Clefts Genetics (Mitchell et al., 2002). During the enrollment of these families, one family was identified with one individual who was affected with non‐syndromic bilateral cleft lip and palate (deceased), which met the initial qualification of the family for the study, and his nephew with bilateral cleft lip and palate as well as ectrodactyly (bilateral split hand and split foot malformation). The current study involves this specific family. The paternal uncle with cleft lip and palate, who died in childhood, was reported to have no other clinical abnormalities; however, he was not available for examination. Upon clinical examination, the 15‐year‐old affected nephew was found not to have any symptoms of ectodermal dysplasia, but he did have redness of his eyes and reported frequent, chronic tearing. He was developmentally normal and had no other apparent clinical symptoms. The deceased affected uncle had between 10 and 15 unaffected siblings (including the affected nephew's father, the exact number of siblings disguised to protect the family's privacy). The affected nephew had between six and 10 unaffected siblings (exact number disguised to protect the family's privacy). The affected nephew's parents were reported to be related, but the family did not specify the exact relationship of his mother to his father.

### 2.2 Whole‐exome sequencing (WES)

Genomic DNA was extracted from EDTA‐treated blood, as described by Bellus et al. (1995). WES was performed in the patient plus his parents and two unaffected siblings using the TruSeq DNA Sample Preparation v2 method (Illumina, San Diego), followed with Illumina's TruSeq Exome Enrichment Kit protocol and sequenced using the Illumina HiSeq2000 with version 3 chemistry to a depth of at least 40 million paired‐end 100 base reads for each sample. Image analysis and base calling were performed with default parameters using Illumina Genome Analyzer Pipeline software (RTA version 1.17.20 and CASAVA 1.8.2).

### 2.3 Alignment and genotype calling

Reads were mapped to NCBI build 37 (hg19) with Novoalign V2.08.02. The aligned lane bam files were merged, sorted, and indexed. Duplicate sequence reads derived from the same original DNA molecule, a polymerase chain reaction (PCR) artifact characterized by molecules having the exact same alignment coordinates for both Read 1 and Read 2, were removed with Samtools. These alignments were stored in BAM format and then fed as input to bam2mpg (http://research.nhgri.nih.gov/software/bam2mpg/index.shtml), which called genotypes at all covered positions using a probabilistic Bayesian algorithm (Most Probable Genotype, or MPG). These genotype calls have been compared against Illumina Human 1 M‐Quad genotype chips, and genotypes with an MPG score of 10 or greater showed >99.89% concordance with SNP Chip data. Sequence bases with a quality score less than 20 (Q20) were ignored. Only reads with mapping quality greater than 30 were included for the variant calling.

### 2.4 Post‐Calling quality control

Genotypes were zeroed out for read depth < 10, genotype quality (GQ) < 10, or a GQ to read depth ratio of <0.5 in Golden Helix SVS v7. Mendelian inconsistencies were identified and examined as candidate de novo mutations. Candidate recessive genes were identified by classifying all variants which were heterozygous in both parents and homozygous in the patient but heterozygous or homozygous for the common allele in the other unaffected offspring.

### 2.5 Annotation

The variants were annotated using Annovar (http://annovar.openbioinformatics.org/en/latest/user‐guide/gene/). Several filtering and prioritization steps were applied to reduce the number and identify potentially pathogenic mutations, similar to the methods used in previous studies (Ng et al., 2009, 2010). Missense variants were sorted by the degree of severity of functional disruption prediction using CDPred and annotated using the Combined Annotation Dependent Depletion (CADD). Variants detected in dbSNP (version 137), 1000 Genomes, NHLBI 6500ESP, and HGMD were annotated.

### 2.6 Detection of candidate recessive loci

Candidate recessive loci were identified using custom scripts in R (https://www.r‐project.org/) and available from the authors on request. Briefly, the script filters loci based on mean allele frequencies from 1000 Genomes, NHLBI's Exome Sequencing Project (ESP), and the Exome Aggregation Consortium (ExAC) combined, identify all loci heterozygous in both parents and homozygous for the rare allele in the patient and then filtered this list to remove variants homozygous for the rare allele in unaffected siblings of the patient.

### 2.7 Detection of Mendelian inconsistencies

Mendelian error detection was performed in PLINK (Purcell et al., 2007), and candidate variants were examined in GoldenHelix SVS.

### 2.8 Sanger sequencing

To confirm candidate variants of interest detected by the analyses above, Sanger sequencing was performed on two variants in seven individuals, consisting of the patient (affected with bilateral cleft lip and palate and ectrodactyly), his parents, and four unaffected siblings. Primers were designed with M13 tags attached for the regions of interest in *TP63* and *HLA‐DRB5* (Supplementary Table S1). PCR products were generated using the KAPA2G Fast HotStart ReadyMix kit (KAPA Biosystems), 2 μM primer, and 2.5 ng genomic DNA. PCR products were treated with ExoSAP‐IT (Affymetrix), and these treated products were used in BigDye Terminator v3.1 Cycle Sequencing reactions (Applied Biosystems) with 10 μM M13 forward and reverse primers, followed by Sanger sequencing on an ABI 3730xl DNA Analyzer (Applied Biosystems). Sequence tracings were analyzed with Sequencher (Gene Codes) software.

### 2.9 Allele‐specific cloning

Due to the difficulty in determining the correct genotype in the patient in the *HLA‐DRB5* sequence data, allele‐specific cloning and sequencing were performed in the patient and both parents. The CloneJET PCR Cloning kit (ThermoScientific) was used to ligate PCR products into the pJET1.2 vector, and they were transformed using competent *E. coli* cells. Direct PCR of colonies was performed with the same *HLA‐DRB5* primer set used in the original PCR, followed by ExoSAP‐IT treatment, BigDye reactions, and Sanger sequencing, as detailed in the section above.

### 2.10 Zebrafish husbandry and ethics statement

All zebrafish experiments were performed in compliance with the National Institutes of Health guidelines for animal handling and research using an Animal Care and Use Committee (ACUC) approved protocol G‐05‐5 assigned to RS. Wild type (WT) zebrafish strain TAB5 was used for all experiments. Zebrafish husbandry and embryo staging were performed as (Westerfield, 2007).

### 2.11 Generation of *tp63* mutants and genotyping

Two single guide RNAs (sgRNAs) targeting exons 5 (GGATGGCAGGTGATGGAGAG) and 6 (GTATGACTGCACCCTGGGGT) of tp63 (Ensembl transcript ID: ENSDART00000127965.4) were designed using the ‘ZebrafishGenomics’ track on the UCSC Genome Browser. Synthesis of target oligonucleotides (Integrated DNA Technologies), preparation of mRNA, microinjections, CRISPR‐STAT to evaluate sgRNA activity, and mutant generation were carried out as described previously (Carrington et al., 2015; Varshney et al., 2015, 2016). Primers used for screening and genotyping by fluorescent PCR were as follows: E5‐Fwd (5′‐ GCTTCTCAACAGCATGGATC) and E5‐Rev (5′‐ TCCAGGTTGCAGATTTGGC), E6‐Fwd (5′‐ CTCCACAGAGTTGAAGAAGC), and E6‐Rev (5′‐ CATTGAACTCTCTGCTCAGC). M13F adapter (5’‐TGTAAAACGACGGCCAGT) was added to the 5′ end of each forward primer, and PIG‐tail (5’‐GTGTCTT) was added to the 5′ end of each reverse primer as described (Sood et al., 2013).

### 2.12 Time lapse imaging

Embryos were immobilized in 1× tricaine and images were acquired every 5 min for a 10 h period using a Leica M125 microscope equipped with an MC170HD camera and the Leica Application Suite (LAS) V4.4. Post‐processing of images was done within the LAS software.

### 2.13 Microinjections of mRNA for rescue of phenotype

The following clones were obtained in pBluescriptII for zebrafish *tp63* (NM_152986.1) and human *TP63* (NM_003722.4) (Genescript). Plasmid DNA was digested with XhoI and mRNA was synthesized using a T7 message machine kit (Ambion). Following transcription, polyA tailing was performed, and RNA was purified by LiCl precipitation. Injection of mRNA (100 pg to 1 ng) into WT embryos was carried out to determine the appropriate dose. Injections (500 pg) of human or zebrafish mRNA were then performed in embryos from in‐crosses of *tp63* ^*+/−*^ fish. Embryos were observed at 72 h post‐fertilization (hpf) and 78 hpf for phenotype and collected for genotyping.

## 3 RESULTS

Before quality control, there were a total of 185,474 SNVs and 18,058 INDELs. After applying quality control filters, variants were dropped for being monomorphic or where all individuals were heterozygous. There were 11,905 INDELS and 137,989 SNVs available for analysis after all filtering steps.

Using a mean allele frequency calculated from the 1000 Genomes Project, the NHLBI Exome Sequencing Project, and the Exome Aggregation Consortium, loci were filtered and were only retained if their mean minor allele frequency was less than or equal to 10%. Thirty‐four loci were homozygous for the minor allele in the patient but heterozygous in the parents and homozygous for the major allele or heterozygous in the unaffected siblings. None of these 34 variants were in known cleft genes, and none of the genes identified were good candidates for orofacial clefting by functionality. The complete list of variants can be found in Supplementary Table S2.

Examining Mendelian inconsistencies revealed 28 candidate de novo events, of which only two were well supported by examination of the alignment and had enough biological plausibility for follow‐up. Details of all 28 loci are listed in Table 1.

**TABLE 1:** Candidate de novo mutations identified in the patient's whole‐exome sequence data, with the two variants selected for follow‐up in bold text.

Chromosome	Position	Gene
1	169,572,298	SELP
2	178,562,139	PDE11A
2	179,578,713	TTN
**3**	**189,585,695**	**TP63**
3	195,506,370	MUC4
3	195,506,606	MUC4
5	140,209,830	*PCDHA* gene cluster
5	154,287,346	GEMIN5
6	32,489,766	HLA‐DRB5/HLA‐DRB1
**6**	**32,489,791**	**HLA‐DRB5/HLA‐DRB1**
6	32,489,792	HLA‐DRB5/HLA‐DRB1
6	32,489,795	HLA‐DRB5/HLA‐DRB1
6	32,489,796	HLA‐DRB5/HLA‐DRB1
8	22,146,113	PIWIL2
10	102,766,505	LZTS2
11	55,110,761	OR4A16
12	6,422,337	PLEKHG6
12	6,437,012	PLEKHG6
12	53,343,163	KRT8/KRT18
12	53,516,993	SOAT2
14	50,906,782	MAP4K5
14	105,180,706	INF2
15	22,369,200	OR4M2
16	30,035,399	C16orf92
19	1,010,782	TMEM259
19	3,543,310	MFSD12/C19orf71
19	17,049,254	CPAMD8
20	23,016,819	SSTR4

**FIGURE 1:**
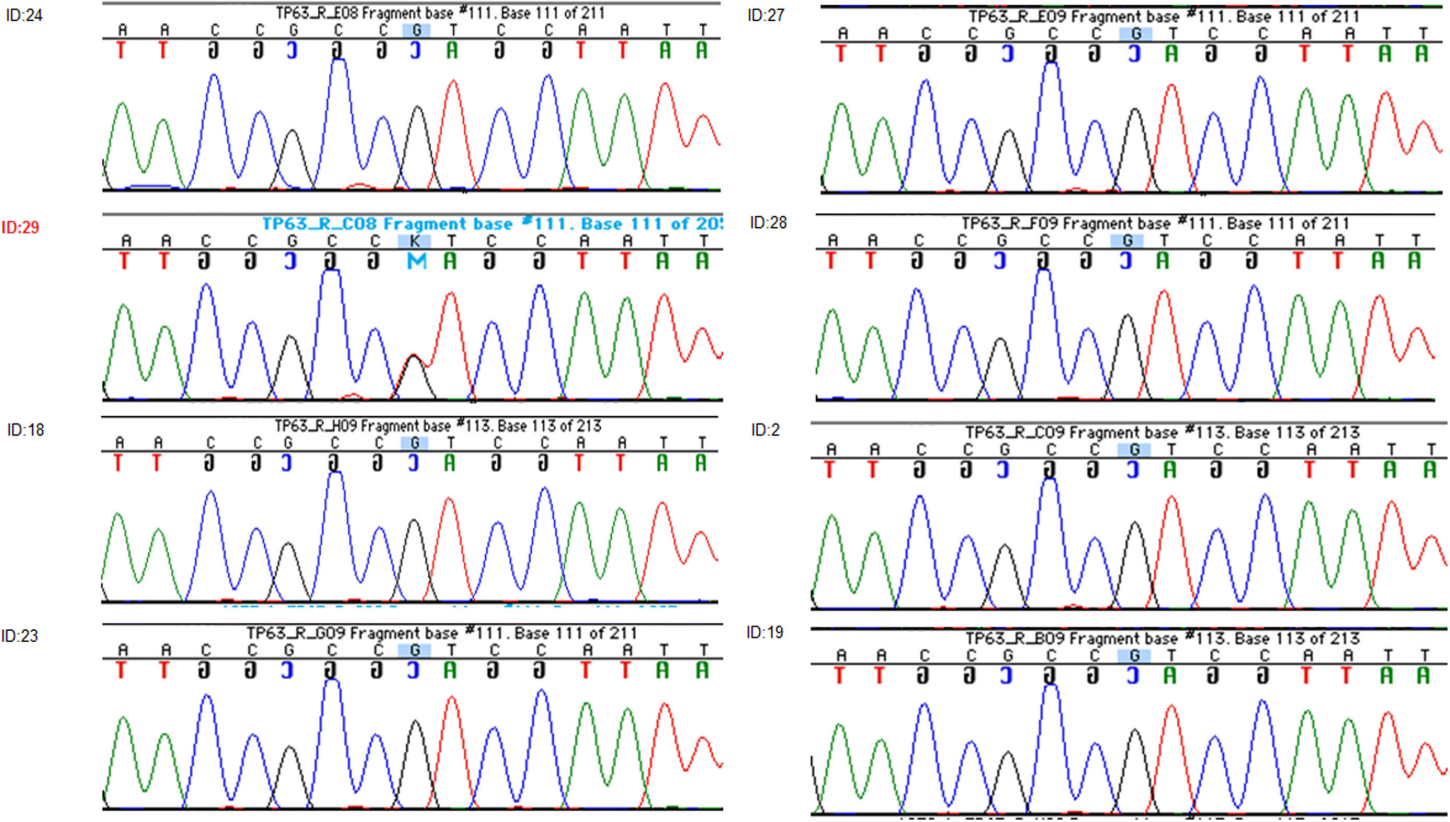
Sanger sequencing results of *TP63* mutation in all available family members.

The most interesting of the de novo candidates was a non‐synonymous, single base substitution in *TP63* (c.956G > T, p.Arg319Leu). Examination of the reads in Golden Helix Genome Browse showed that the call had good coverage and was present in reads in both directions, consistent with a heterozygous mutation. Sanger sequencing in the entire pedigree confirmed this was a likely true de novo mutation (Figure 1). The PHRED‐scaled CADD score for this mutation was 31, placing it in the class 5 “pathogenic” range. Examination of whole‐genome or whole‐exome sequencing of 37 additional individuals with non‐syndromic oral clefts from other Syrian oral cleft families (Bureau et al., 2014) revealed only two coding variants in *TP63*; rs140508531, a rare synonymous SNV previously seen in ExAC and ESP. The other was a synonymous SNV not reported in any databases. Genotypes for this variant for each individual called from the Sanger sequencing can be found in Table 2. We also examined exome and genome sequences from other populations of non‐syndromic oral cleft patients (populations details and numbers can be found in Table 3) and found two more synonymous variants in *TP63*; one had never been seen in any of the online databases, the other was rs33979049, an uncommon SNV seen in 1000 Genomes at a minor allele frequency (MAF) of between 1 and 5%, depending on population. In evaluating the de novo c.956G > T, p.Arg319Leu mutation in our patient, one should note that leucine is considerably smaller than arginine which means that the amino acid is not in the correct position to make the hydrogen bonds with E239, G315 and M316. Leucine is also more hydrophobic and its charge is neutral, unlike the positively charged arginine. R319 forms a salt bridge with E239 so the difference in charge will disturb the ionic interaction necessary for this process. As expected, all of these differences between the two residues would likely have significant impact on the conformation and function of the protein (Venselaar et al., 2010) (See Supplementary Figures S1 and S2).

**TABLE 2:** Genotype results of *TP63* and HLA‐DRB5 Sanger sequencing, with the patient highlighted in bold text.

Relationship	Individual ID	*TP63* genotype	HLA‐DRB5 genotype (rs41550412)
Paternal Grandparent	2	G/G	T/C
Father	18	G/G	T/G
Mother	19	G/G	C/C
**Patient**	**29**	**G/T**	**C/G**
Sibling 1	23	G/G	T/C
Sibling 2	24	G/G	T/C
Sibling 3	27	G/G	T/C
Sibling 4	28	G/G	T/C

**TABLE 3:** Number of individuals affected with non‐syndromic oral clefts* and with available DNA sequence data.

Population	Number of individuals (families) with WES^a^	Number of individuals (families) with WGS^a^
Syrian	16 (8)	37 (14)
Indian	26 (12)	37 (19)
Filipino	22 (11)	78 (18)
German	38 (19)	0
Taiwanese	2 (1)	3 (1)
European‐American	3 (1)	4 (1)
Chinese	2(1)	
Guatemalan		3 (2)
Singaporean		5 (3)

^a^ Multiple affected individuals were sequenced from multiplex families; 6 Syrian individuals from 2 families were sequenced with both WES and WGS. They are only counted here in the WGS totals.

**FIGURE 2:**
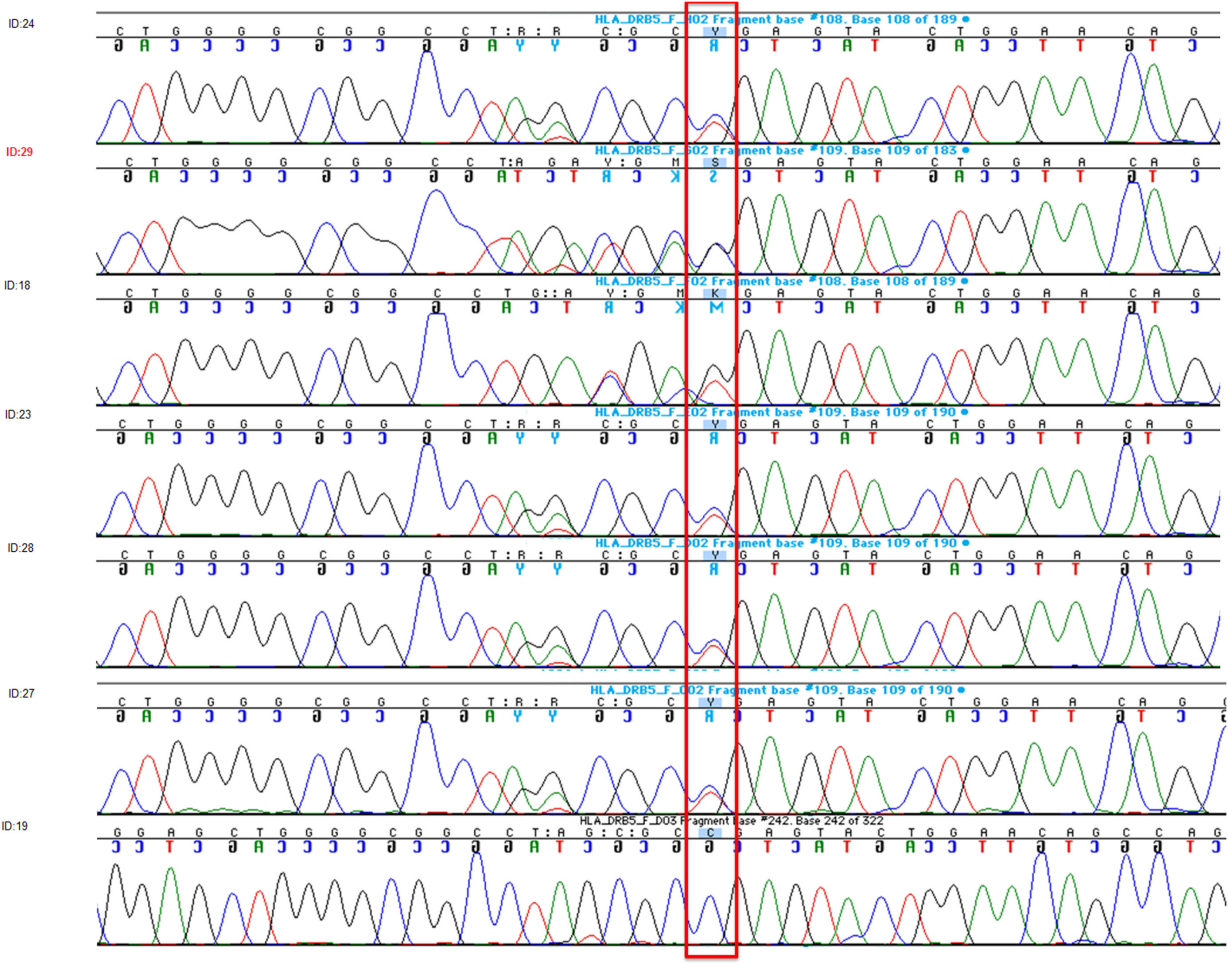
Sanger sequencing results of HLA‐DRB5 variant in all available family members.

**FIGURE 3:**
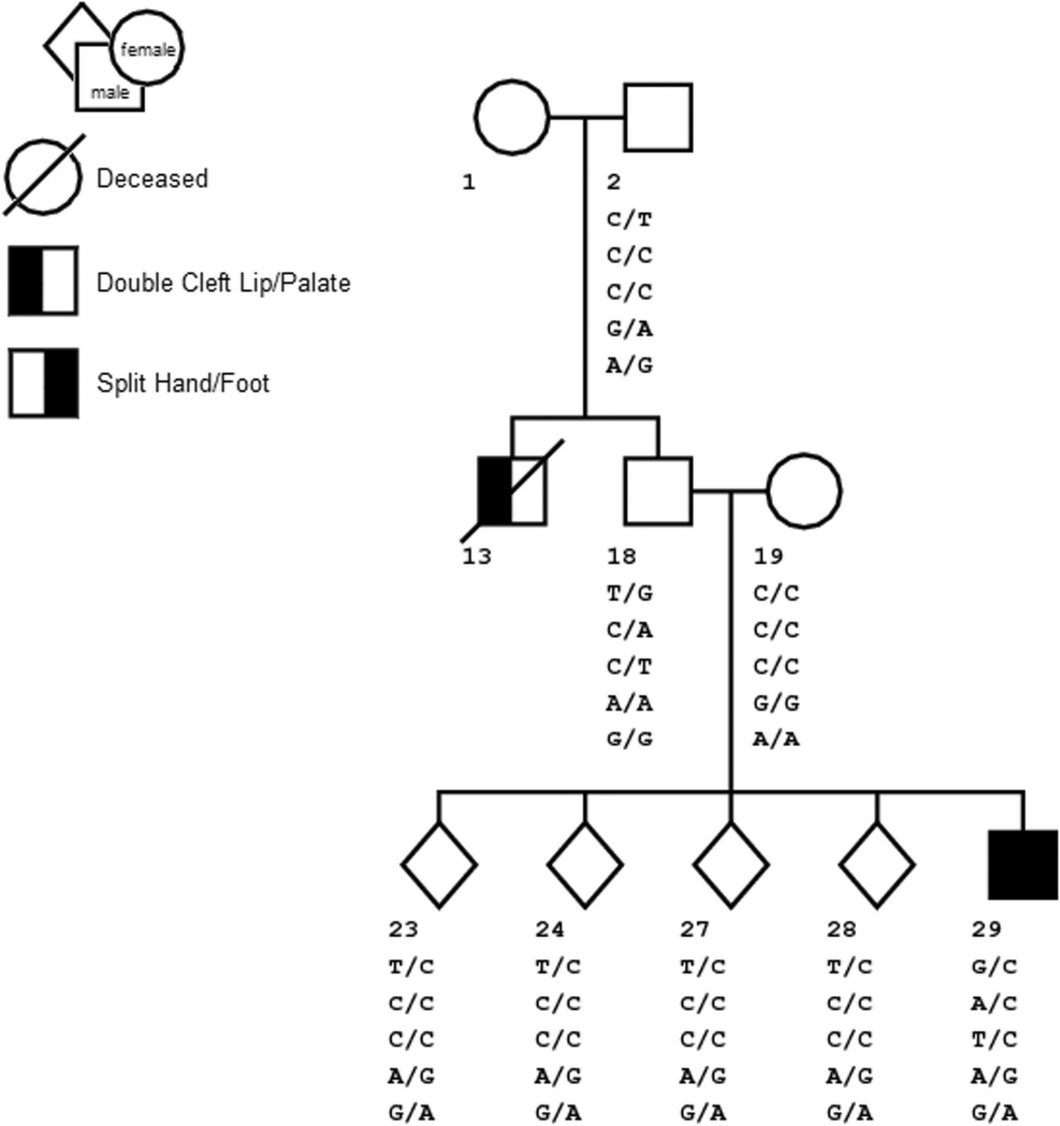
HLA‐DRB5 Genotypes from Sanger Sequencing for all available family members.

The second putative de novo candidate was a third allele in the known SNP, rs41550412, in *HLA‐DRB5*. Annotation of the variant in dbSNP and CADD and examination of the reads in Genome Browser suggested that this was a tri‐allelic SNP, has a CADD score of 4.4 and 4.0 for the G and C alleles, respectively, and therefore not in the likely pathogenic range and might be segregating normally in the family. Sanger sequencing confirmed this hypothesis (Table 2) and showed this family was segregating four additional non‐synonymous variants in this gene (Figure 2) that had not been correctly captured by the WES due to the coverage level and allele‐specific read imbalance at this location. Allele‐specific cloning and Sanger sequencing of both parents and the patient was performed to clarify the inheritance of 5 loci in this gene (Figure 3, Supplementary Table S3). The patient had a unique combination of all five variants (Figures 2 and 3) that any of his four genotyped unaffected siblings did not share. However, none of these variants are rare, so it is probable that this is a chance finding. Nevertheless, compared to the other non‐syndromic oral cleft sequence data we have generated (Table 3), none of the additional exome or genome sequences examined shared any of these variants.

We also examined 67 candidate genes (Supplementary Table S4) for recessively inherited variants in this family. However, no coding variation in these genes produced unique genotypes in the patient compared to his siblings.

### 3.1 Generation of *tp63* ^−/−^ zebrafish mutants

**FIGURE 4:**
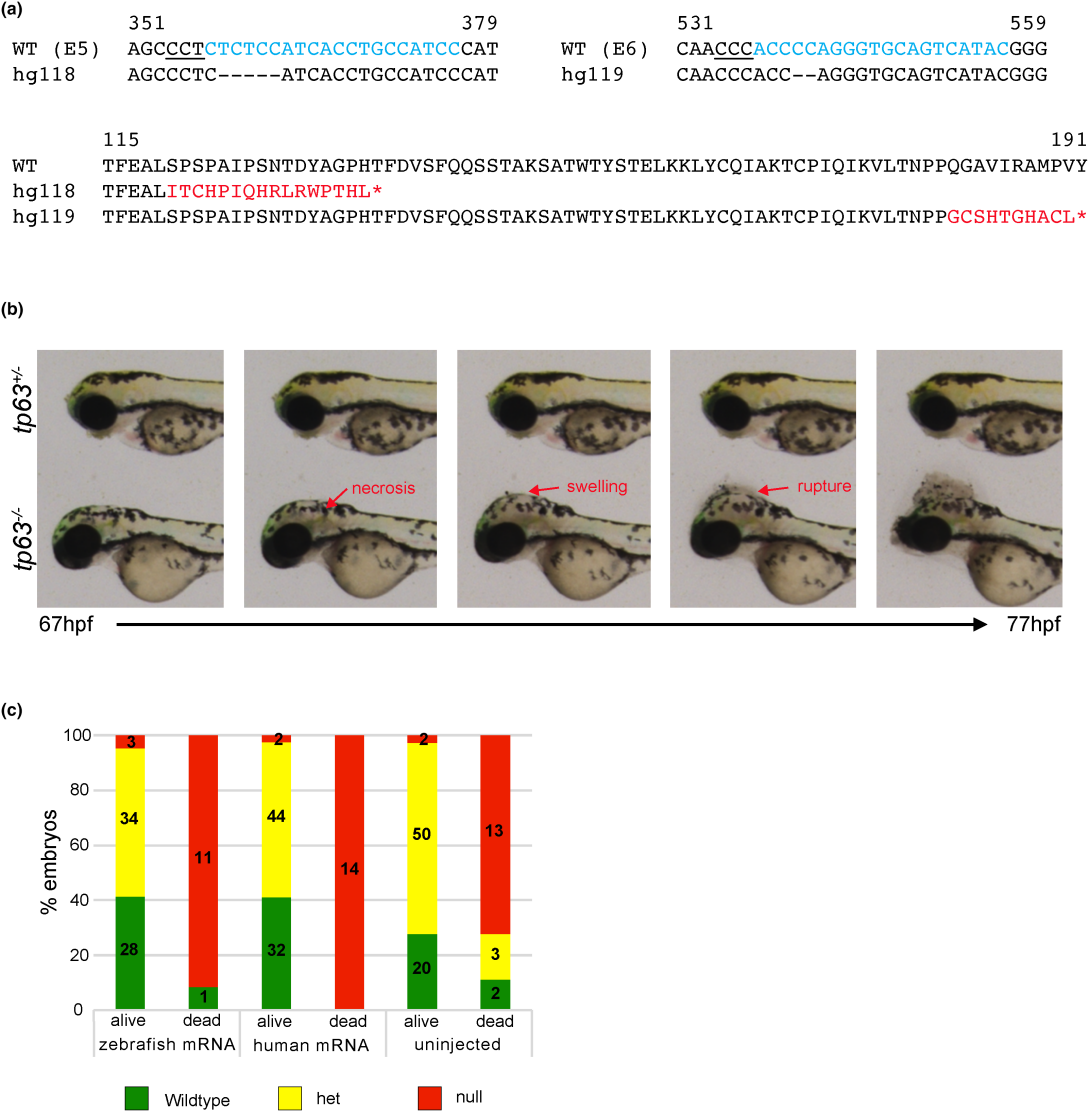
Generation and characterization of *tp63* knockout mutants. (a) Top panel: Nucleotide sequences of CRISPR target regions in exons 5 and 6 with sgRNAs marked in cyan, PAM sites underlined, and deleted nucleotides in the mutant alleles marked by dashes. Bottom panel: An alignment of WT and mutant proteins with the frameshift and premature stop codons marked in red. (b) Phenotype of the *tp63* ^*−/−*^ embryos shown as images at different time points from a time lapse video taken from 67 to 77 hpf. Images of a heterozygote sibling are shown for control. At 67 hpf the *tp63* ^*−/−*^ embryo is indistinguishable from its heterozygous sibling. Shortly after necrosis within the head begins followed by swelling and rupture as marked by red arrows. (c) Data from rescue experiments by injections of 500 pg of zebrafish or human *tp63* mRNA shown as bar graphs. Embryos were observed for phenotype between 72–78 hpf, separated into dead or alive groups and genotyped. Different groups of embryos that were scored are shown on the X‐axis and percentages of embryos of different genotypes are shown on the Y‐axis.

To validate the role of *tp63* in this proband's phenotype, we generated *tp63* knockout fish lines using CRISPR/Cas9 technology. To ensure that the mutation affects all known isoforms, we selected gRNAs to exons 5 and 6 that are common to all isoforms (Supplementary Figure S1). Two mutant alleles (del 5 bp in exon 5 and del 2 bp in exon 6), predicted to cause frameshifts with premature truncations of the protein, were selected for the study (Figure 4a). These mutant alleles have been given designations from the Zebrafish international Resource Center (http://zfin.org/action/feature/line‐designations) as *tp63* ^*hg118*^ (c.358_362delTCTCC; p.Ser120Ilefs*17) and *tp63* ^*hg119*^ (c.540_541delCC; p.Gln181Glyfs*11).

### 3.2 Loss of *tp63* leads to necrosis and rupture of head at 3 dpf

To determine the effect of loss of function of *tp63* on development, we performed pairwise crosses of heterozygous fish for each mutant allele separately and observed their progeny for morphological phenotypes. Homozygous mutant embryos for both alleles displayed similar morphological phenotype and hence are collectively referred to as *tp63* ^−/−^. The *tp63* ^−/−^ embryos were indistinguishable from their WT and heterozygous clutch mates for the first two days of development. At 3 dpf, they displayed a rupture of the head followed by death (Figure 4b). Time lapse imaging of live embryos from 68 to 77 hpf revealed that about 2–3 h prior to head rupture, necrosis and swelling occurs in the head region of mutant embryos (Figure 4b). Therefore, we were unable to evaluate jaw and fin development in *tp63* ^−/−^embryos to validate its role in the patient's phenotype. Although the patient is heterozygous for the *TP63* mutation, *tp63* ^*+/−*^ zebrafish embryos appeared morphologically identical to their *tp63* ^+/+^ siblings and survived to adulthood.

### 3.3 Rescue of embryonic phenotype by injection of zebrafish or human mRNA

We hypothesized that if the *tp63* ^−/−^ phenotype can be rescued by WT *tp63* mRNA, then we could evaluate the patient variant for its effect on *Tp63* function using the rescue assay. We performed dose response curve by injecting either human or zebrafish WT *tp63* mRNAs into WT embryos and monitoring their viability and morphological phenotype (data not shown). Subsequently, 500 pg of zebrafish or human mRNA was injected into embryos from an in‐cross of *tp63* ^*hg118/hg118*^ fish. We did not observe any phenotypic or survival improvements in the injected *tp63* ^*hg118/hg118*^ embryos (Figure 4c), indicating that the phenotype is too severe to be rescued by complementation by WT mRNA. Therefore, the rescue assay was not applicable for validation of the patient variant.

## 4 DISCUSSION

Mutations in *TP63* are known to cause a number of different malformation syndromes, which include orofacial clefting or limb malformations in their phenotypic presentation, including Ectrodactyly, Ectodermal Dysplasia and Cleft Lip/Palate Syndrome 3 (EEC3), Split Hand/Foot Malformation 4 (SHFM4), Hay–Wells Syndrome, ankyloblepharon‐ectodermal defects‐cleft lip/palate (AEC) syndrome (including Rapp–Hodgkin syndrome), and Acro‐dermato‐ungual‐lacrimal‐tooth (ADULT) syndrome. The phenotype demonstrates considerable phenotypic variability which can include hypohidrosis, nail dysplasia, sparse hair and tooth abnormalities, hypopigmentation, hypoplastic breasts and with or without hypoplastic nipples, hypospadia, and lacrimal duct obstruction as well as cleft lip/palate and split‐hand/foot malformation/syndactyly. Incomplete penetrance has also been observed in a small number of individuals and pedigrees (Amiel et al., 2001; Spranger & Schapera, 1988). Genotype–phenotype analyses have shown the link between the various clinical presentations of *TP63* related disorders (Alves et al., 2015; Harazono et al., 2022). The R319L mutation reported here appears novel, with no recorded instances in public variation or clinical databases. Two other mutations have been reported at the same codon – the SHFM4 mutation 1 bp upstream (rs121908839, c.955C > T, p.Arg319Cys) (van Bokhoven et al., 2001) and a case report of EEC/LM/ADULT in a Chinese family (c.956G > A, p.(Arg319His) (Otsuki et al., 2020) changing the same nucleotide base as our R319L mutation.). Both produce different amino acid changes which provides strong evidence that mutating this amino acid is deleterious. These mutations in exon 7 of *TP63* are in the DNA‐binding domain of the protein and are highly evolutionarily conserved. However, it is not completely clear whether the R319L mutation discovered here is only responsible for the ectrodactyly or whether it is also responsible for the oral cleft seen in the patient. The R319C and R319H mutations are so far known only to cause ectrodactyly (Ianakiev et al., 2000; Otsuki et al., 2020; van Bokhoven et al., 2001) and not orofacial clefting although the R319H mutation was associated with missing teeth and R319C had tooth abnormalities. It cannot be ruled out that the patient's unique compound heterozygous mutations in *HLA‐DRB5* are causing the oral cleft and *TP63* is responsible for the ectrodactyly phenotype. Although *HLA‐DRB5* has not been associated with orofacial clefting or limb malformations, it is one of the *HLA‐DRB* cluster of genes in the HLA region of chromosome 6 and a paralog of the known oral cleft gene *HLA‐DRB1* (Doxiadis et al., 2012), some transcripts of which may overlap. Given the affected paternal uncle and the consanguinity of the patient's parents, there is the possibility that these are two co‐occurring but independent features. There is considerable heterogeneity in the phenotypic presentation of *TP63* mutations as demonstrated by Harazono et al, so either possibility cannot be completely excluded although it seems more likely that *TP63* is responsible for the entire phenotype as it more closely fits with the features of EEC.

Whole‐exome sequencing has been used to great effect in finding causal mutations in patients with genetically heterogeneous diseases. Our study has identified a previously unknown mutation in *TP63*, a gene known to be associated with both oral clefts and ectrodactyly. However, sequencing alone cannot determine exactly which parts of the observed phenotype are caused by this mutation. It is possible that the orofacial cleft may have another etiology since the patient's uncle also had an oral cleft independent of the de novo *TP63* mutation seen here. However, given the prevalence of oral clefts in the Syrian population and the known consanguinity in this pedigree, the uncle's cleft may be etiologically distinct from his nephew's. Unfortunately, determining causation becomes more complex because the uncle died in childhood.

The results from our zebrafish experiments were intriguing, if not definitive. We were unable to do functional validation of the patient variant due to severity of the knockout phenotype and inability to rescue it by mRNA injections. A recent study has demonstrated that *tp63* is required for ectoderm specification during zebrafish development (Santos‐Pereira et al., 2019). Interestingly, *tp63* mutant embryos used in that study (Santos‐Pereira et al., 2019) died prior to the stage at which head necrosis was observed in our mutant lines. The head necrosis is a novel finding in our study. These phenotypic differences between different mutant alleles are most likely due to their effects on the multiple isoforms of *tp63*. Consistent with the Santos‐Pereira et al. findings, the heterozygous embryos (*tp63* ^*+/−*^) in our study did not display any morphological phenotypes. Closer examinations of their developing ethmoidal plate are required to rule out any subtle phenotypes in *tp63* ^*+/−*^ larvae.

In‐depth functional analysis is both expensive and time‐consuming, but there is only so far computational, and statistical methods can take us, and additional in vivo studies may be required. Specifically, generation of a zebrafish or other model organism with this exact variant in *TP63* (c.956G > T, p.Arg319Leu) using knock‐in or base editing is required for further evaluation of its role in the proband's phenotype.

## 5 CONCLUSIONS

This study represents the first study of its kind in Syria. The *TP63* novel variant identified here is an excellent candidate for being the cause of the bilateral ectrodactyly in this patient, which has important clinical implications, suggesting that any change in this codon may have severe consequences developmentally. It is also an excellent candidate for being the cause of the patient's oral cleft since this fits the pattern of *TP63*‐Related Disorders (Harazono et al., 2022) and similar phenotypes observed in individuals with other mutations in the same codon of *TP63*. However, it is possible that this de novo variant is not the cause of the patient's bilateral cleft lip and palate given his uncle's cleft lip and palate and biological relationship of his parents. The family structures in this region of the world can be challenging and the incidence of oral clefts is high. Studying oral clefts is of great significance to global health because it is common, and the impact on child growth and development is substantial. Whereas corrective surgery in early childhood can mitigate most of the effects of the disorder, this surgery is out of reach for many living in low‐ and middle‐income countries, to devastating effect. Understanding the ways in which genes influence the development of the trait is an essential step to determining mechanisms and potential preventive options in the future for individuals at high risk of having a child with an oral cleft. Even de novo variants, as presented here, can provide those insights.

## AUTHOR CONTRIBUTIONS

Claire L. Simpson performed data cleaning and analyses in the human data and wrote the bulk of the manuscript. Danielle C. Kimble and Settara C. Chandrasekharappa performed the Sanger sequencing and genotype calls, commented and revised the manuscript. NISC Comparative Sequencing Program performed the whole‐exome sequencing and bioinformatics processing. Khalid Alqosayer, Ghiath Al‐Souqi and Hasan Albacha‐Hejazi contributed to study design, recruited patients and commented on the manuscript. Emily Holzinger assisted with data analyses. Blake Carrington, John McElderry and Raman Sood performed all of the zebrafish experiments and wrote the relevant sections of the manuscript. Joan E. Bailey‐Wilson donated resources from the Intramural Program at NHGRI and contributed to funding applications, study design, assisted with statistical analyses and revised the manuscript.

## FUNDING INFORMATION

This work was supported in part by the Intramural Program of the National Human Genome Research Institute, National Institutes of Health, and grants R01 DE014581 (International Genetic Epidemiology of Oral Clefts) and U01 DE020073 (Oral Clefts: Moving from Genome‐Wide Studies Toward Functional Genomics).

## CONFLICT OF INTEREST STATEMENT

The authors have no competing interests to declare.

## ETHICS APPROVAL AND CONSENT TO PARTICIPATE AND FOR PUBLICATION

The study was approved by the Institutional Review/Ethics Boards of the National Human Genome Research Institute, NIH (USA), and the Ibn Al‐Nafees Hospital (Damascus, Syrian Arab Republic). All study participants provided written informed consent (in Arabic), and the study followed the tenets of the Declaration of Helsinki.

## Supporting information


Table S1

**Figure S1** Schematic of the genomic organization of all known *tp63* isoforms taken as screen shot from ensembl. Target exons 5 and 6 are boxed and shared by all isoforms.

## Data Availability

All data generated or analyzed during this study are included in this published article (and its supplementary information files) where possible. The informed consent forms and the protocol on file with the Institutional Review Board at the NIH both guarantee the pedigrees will never be published to protect the privacy of the study participants because these pedigrees are readily identifiable given the rarity of such multiplex oral cleft families. Therefore, only a redacted and disguised version of the pedigree is included and full genetic data cannot be shared.
