# Supplementary figures and images for: A novel de novo TP63 mutation in whole‐exome sequencing of a Syrian family with Oral cleft and ectrodactyly
Source: Mol Genet Genomic Med. 2023 Apr 18;11(8):e2179. doi: 10.1002/mgg3.2179 (PMC10422068; doi:10.1002/mgg3.2179)

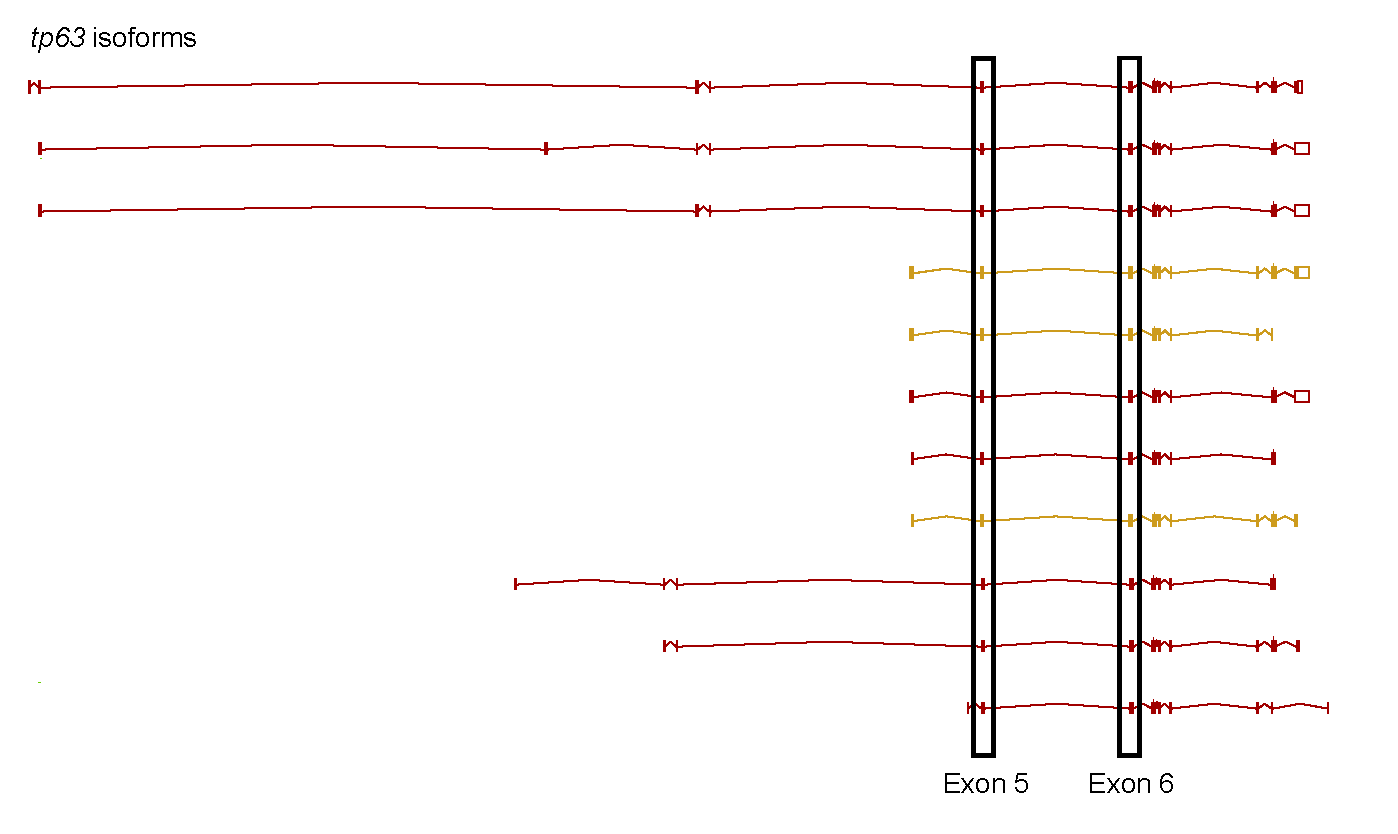

Supplement: Supplementary file 2 — Figure S1 Schematic of the genomic organization of all known tp63 isoforms taken as screen shot from ensembl. Target exons 5 and 6 are boxed and shared by all isoforms. [file MGG3-11-e2179-s001.png]
